# Slippery Consequences: An Eight-Year Review of Temporary Ice Rink-Related Hand and Wrist Injuries at a Major Trauma Centre

**DOI:** 10.7759/cureus.92210

**Published:** 2025-09-13

**Authors:** Chan Khin, Adeife Erinfolami, Chido Nwankwo, Olive Kyaw, Amr Elfiky, Prashanth D'Sa

**Affiliations:** 1 Trauma and Orthopaedics, University Hospitals Sussex NHS Foundation Trust, Brighton, GBR; 2 Orthopaedic Surgery, University Hospitals Sussex NHS Foundation Trust, Brighton, GBR

**Keywords:** covid-19 pandemic, distal radius fractures, hand injuries, ice rink, ice skating injuries, wrist injuries

## Abstract

Introduction

Temporary ice rinks are popular seasonal attractions but pose significant risks of injuries due to variable surface conditions, high user density, and limited safety infrastructure. Distal radius fractures are well recognised in this context, yet long-term data describing their burden and management remain limited. This study aimed to characterise injury patterns and assess their impact on imaging, management, and surgical workload.

Methods

A retrospective review of a prospectively maintained database was conducted at a major trauma centre in the United Kingdom, covering temporary ice-skating seasons from 2017 to 2024. All patients aged 18 years or over who sustained hand and wrist injuries attributable to ice-skating activities were included; patients under 18 years were excluded. Data were extracted from the virtual fracture clinic database. Descriptive statistics summarised demographics, injury patterns, imaging use, and management. Temporal trends were evaluated using Poisson regression, comparing pre-pandemic (2017-2019), pandemic (2020), and post-pandemic (2021-2024) seasons.

Results

A total of 117 patients met the inclusion criteria, comprising 97 females (82.9%) and 20 males (17.1%), with a mean age of 40 ± 15 years (range 18-74). Of these, 105 patients sustained 120 radiographically confirmed bony injuries, most commonly distal radius fractures (n = 81, 67.5%). Other injuries included ulnar (n = 11), radial shaft (n = 7), carpal bones (n = 13), phalanges (n = 5), and metacarpals (n = 3). Twelve patients (10.3%) had isolated soft-tissue injuries. Fourteen patients (12%) required surgery, predominantly open reduction and internal fixation of distal radius fractures. Annual case numbers declined sharply during the 2020 season (IRR = 0.19, 95% CI 0.06-0.58, p = 0.004) but rebounded post-pandemic. The mean injury incidence was ~1.7 per 10,000 skating visits per season.

Conclusions

Temporary ice rink-related hand and wrist injuries represent a recurrent seasonal burden on trauma services, with distal radius fractures comprising the majority of cases and frequently requiring surgery. These findings highlight the importance of preventive strategies, including public education, the promotion of protective equipment, and enhancement of rink safety standards.

## Introduction

Temporary ice rinks have become increasingly popular in urban settings, offering seasonal recreational opportunities but posing significant injury risks due to factors such as variable surface quality, high user turnover, and limited safety infrastructure [1]. Ice skating injuries span a broad epidemiological spectrum, with studies documenting both recreational and competitive contexts, showing peak incidences during colder months and surges linked to temporary rink operation [2-4]. Although lower limb fractures and head injuries are often highlighted in skating epidemiology [5,6], hand and wrist injuries represent a distinct subset with patterns linked to falls on outstretched hands, injuries from skate blades, and fractures, particularly of the distal radius [7-9]. Clinical presentations vary from simple contusions to complex open injuries requiring operative management, underscoring the need for timely and accurate diagnostic investigations, including radiography and advanced imaging to assess articular involvement or associated soft-tissue damage [10]. Despite calls for injury prevention strategies-including rink design improvements and protective equipment, there remains limited longitudinal evidence characterising these injuries in detail [2].

This study aims to identify the common hand injuries sustained at a temporary ice rink and to evaluate their impact on the hand surgery service in a major trauma centre over an eight-year period. This study seeks to address existing gaps in the literature, with particular emphasis on injury patterns. The primary outcome is to characterise the patterns of hand injuries related to ice rink incidents, while secondary outcomes assess the overall injury burden, including imaging utilisation, initial treatment strategies, follow-up requirements, and management approaches.

## Materials and methods

Study design and setting

This was a retrospective review of a prospectively maintained database from the University Hospitals Sussex NHS Foundation Trust, a major trauma centre in the United Kingdom, covering ice skating seasons from 2017 to 2024. For the purpose of this study, one season was defined as the winter period during which the local temporary ice rink operated (typically early November to late January). Each season is labelled by its starting calendar year (e.g., ‘2020 season’ = November 2020 to January 2021). The hospital is located in close proximity to the rink, and it was assumed that individuals sustaining rink-related injuries would present to the on-site Accident and Emergency (A&E) department, from which referrals were subsequently made via the Virtual Fracture Clinic (VFC) pathway.

Study methodology

Ice rink-related injuries were identified through documentation within the VFC database. Cases were included if the presenting history, at the time of referral from A&E, explicitly indicated that the injury had occurred during ice skating at the local temporary rink.

At our institution, patients presenting to A&E with suspected hand or wrist injuries are referred to the VFC if they meet standard referral criteria, including: (1) radiologically confirmed or clinically suspected fractures requiring orthopaedic follow-up; (2) injuries likely to benefit from specialist hand surgery input (e.g., intra-articular involvement, displacement, or high functional demand); (3) unclear or complex diagnoses requiring specialist review. Minor soft tissue injuries, non-fractures, or injuries definitively managed within A&E (e.g., buddy strapping or simple splinting without need for follow-up) were not routinely referred and are therefore not captured in this dataset.

Inclusion and exclusion criteria

Patients were included if they met all of the following: (1) aged 18 years or older at the time of injury; (2) sustained an injury to the hand and/or wrist; (3) sustained the injury while ice skating at the local temporary ice rink; (4) were referred from A&E to the VFC for orthopaedic assessment and management.

Patients were excluded if they were: under 18 years; sustained injuries to anatomical regions other than the hand or wrist (e.g., elbow, shoulder, or lower limb); or were managed entirely within A&E without referral to the VFC.

This approach may underestimate the overall incidence of ice rink-related hand and wrist injuries in the local population, but ensures that the dataset accurately reflects the caseload managed by our hand surgery service.

Study endpoints

The primary outcome was to analyse the patterns of hand and wrist injuries associated with ice rink incidents. Secondary outcomes included evaluating the overall injury burden, with particular emphasis on imaging requirements and whether patients were managed conservatively or surgically. The findings aim to inform public health messaging and raise awareness of the risks associated with ice rink activities.

Statistical analysis

All statistical analyses were performed using Microsoft Excel and Python (with relevant statistical libraries). Descriptive statistics were used to summarise patient demographics, injury patterns, imaging utilisation, and management approaches. Data are presented as absolute numbers (N) and percentages (%). Continuous variables (e.g., age) are reported as mean ± standard deviation (SD).

To assess temporal trends, seasons were grouped as pre-pandemic (2017-2019), COVID-19 season (2020), and post-pandemic (2021-2024). For regression analyses, COVID-19 was coded as 1 for the 2020 season and 0 otherwise. Poisson regression modelling was used to evaluate annual trends while adjusting for calendar year, with year treated as a continuous predictor and a binary indicator for the COVID-19 season included to estimate its independent effect. Incidence Rate Ratios (IRRs) with 95% confidence intervals (CIs) were calculated. Statistical significance was defined as p < 0.05.

Ethical approval

The UK Medical Research Council and NHS Health Research Authority decision-making tool was used to ascertain whether this project constituted research requiring formal ethical approval [11]. It was confirmed that ethical approval was not required. This study, therefore, constituted a service evaluation project, and local governance approval was obtained.

## Results

Patient data

Over the eight-year study period, 117 patients who sustained hand and wrist injuries while ice skating and were referred to the VFC were included in the study. The cohort comprised 97 females (82.9%, 95% CI 75.8-89.0%) and 20 males (17.1%, 95% CI 11.0-24.2%). The mean age was 40 years (SD ±15), with a range of 18 to 74 years. Injuries were slightly more common on the left hand (53.8%, 63 patients) compared to the right (46.2%, 54 patients).

Primary endpoint

Among the 117 total cases, 105 patients (89.7%) sustained radiographically confirmed 120 bony injuries, while 12 patients (10.3%) were diagnosed with soft-tissue injuries based on clinical and radiographic assessment.

Distal radius fractures were by far the most common injury pattern, accounting for more than two-thirds of all bony injuries. Other fracture types were less frequent and included ulnar, radial shaft, scaphoid, other carpal bones, phalanges, and metacarpals (Table 1).

**Table 1:** Distribution of bony hand injuries sustained at a temporary ice rink (2017-2024) Data are presented as number of fractures (N) and percentage (%) of total injuries. Percentages are calculated relative to the total number of hand and wrist fractures (N=120).

Fracture type	Number of fractures (N)	Percentage (%)
Distal radius	81	67.5
Ulnar	11	9.2
Shaft of radius	7	5.8
Scaphoid	5	4.2
Carpal bones other than scaphoid	8	6.7
Phalanges	5	4.2
Metacarpals	3	2.5

**Figure 1:**
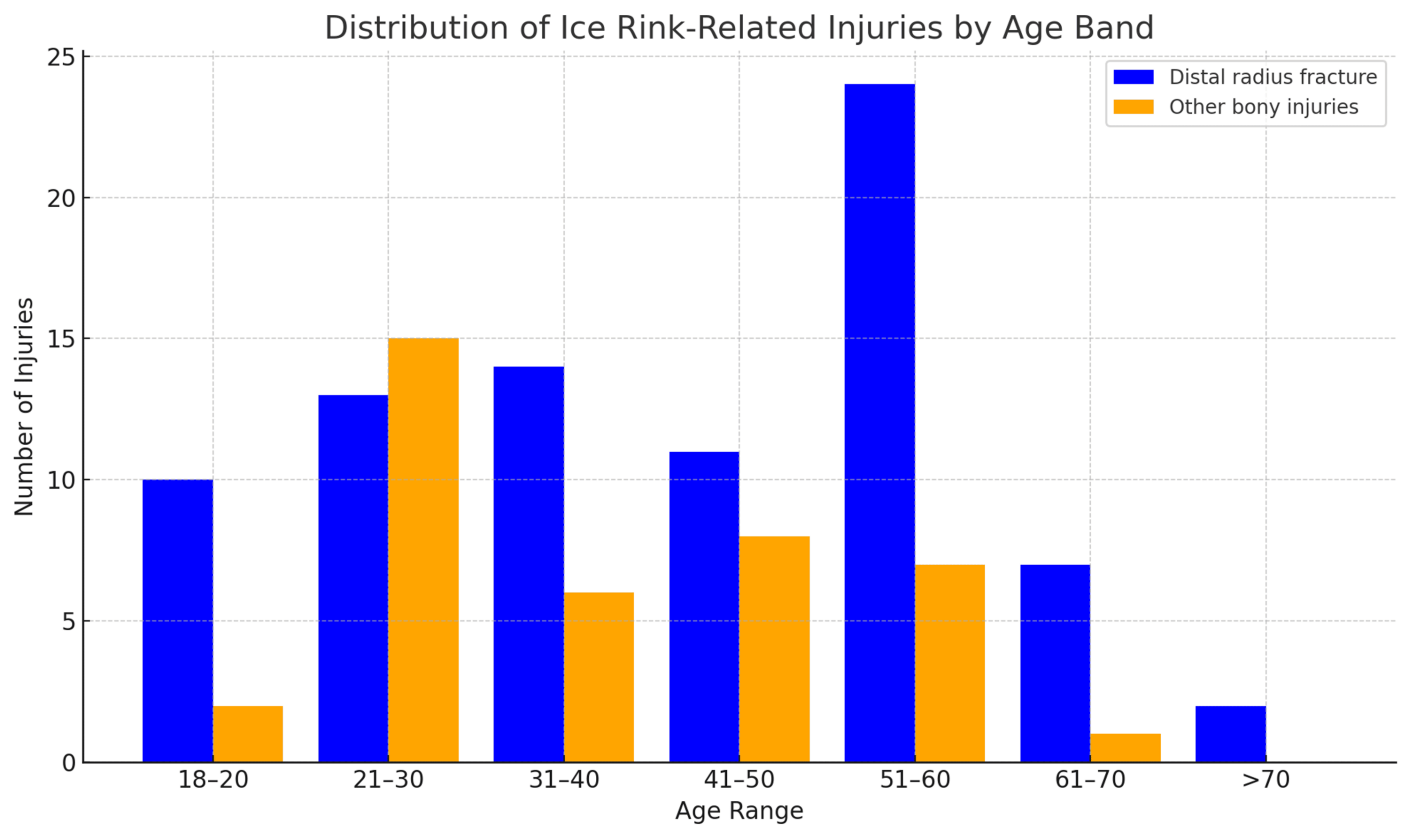
Distribution of ice rink-related hand and wrist injuries across age groups Data are presented as number of injuries (N) within each age band, separated into distal radius fractures and other bony injuries. Descriptive distribution only.

The incidence of distal radius fractures peaked in the 51-60 age group, exceeding all other age bands, whereas other bony injuries showed a more even distribution across younger and middle-aged adults (Figure 1). Notably, the 21-30 age group had the highest proportion of non-distal radius fractures relative to total injuries (Figure 1).

Secondary endpoint

Out of the total cases, 14 patients underwent operative management. Thirteen of these procedures involved open reduction and internal fixation (ORIF) of distal radius fractures. One patient required wound washout, soft tissue extensor tendon repair, and closure due to an open fracture. Radiographic imaging with X-rays was performed in all cases. Computed tomography (CT) scans were conducted in 6 patients, primarily to evaluate fracture patterns (3 cases), assess fracture union (1 case), rule out scaphoid injury (1 case), and confirm fractures not visible on X-rays (1 case). Magnetic resonance imaging (MRI) was performed in 10 patients, mainly to detect scaphoid fractures not visible on X-rays (2 cases), assess distal radius fractures and evaluate scaphoid integrity (5 cases), rule out scaphoid injury (1 case), confirm fractures not visible on MRI (1 case), and investigate soft tissue ligament injuries (1 case).

Trend analysis over time

**Figure 2:**
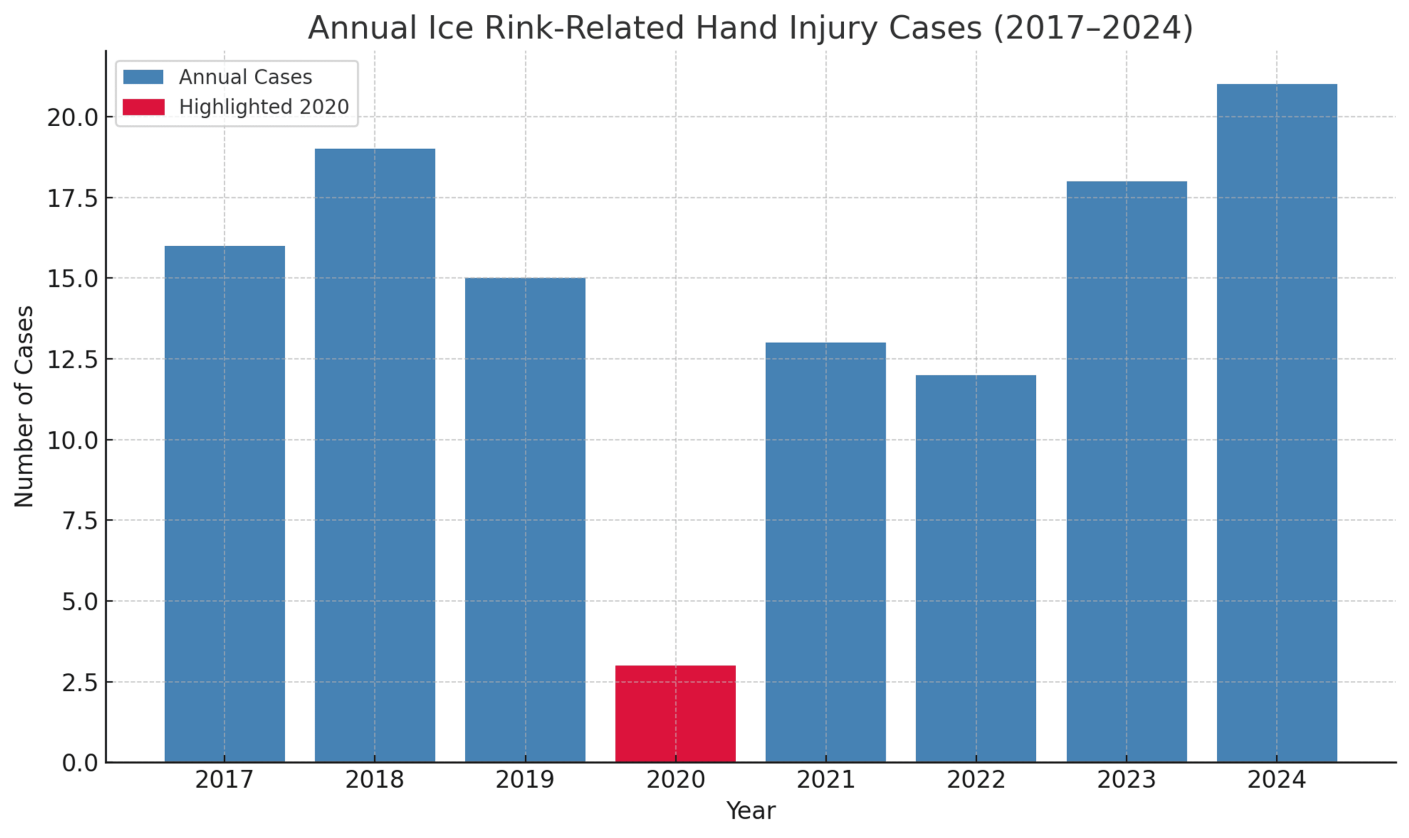
Annual number of ice rink-related hand and wrist injury cases from the 2017 to 2024 ice skating seasons at a major trauma centre Data are presented as raw case counts per season. The red bar highlights the 2020 ice skating season (November 2020 to January 2021), during which a significant reduction in injuries was observed (Poisson regression IRR = 0.19, 95% CI 0.06–0.58, p = 0.004). Statistical significance was defined as p < 0.05.

This study presents a trend analysis of ice rink-related hand and wrist injuries across eight consecutive winter seasons (2017-2024). Annual case numbers varied over the study period, with a notable decline during the 2020 ice skating season (November 2020 to January 2021), when only three injuries were recorded (Figure 2). This sharp reduction temporally aligned with the COVID-19 pandemic, during which public health restrictions led to temporary rink closures and reduced public participation.

While the trend itself may not have direct clinical application for individual patient care, it offers valuable insight into the broader patterns of injury incidence and the resulting demands on trauma services. The mean annual case volume in pre-pandemic years (2017-2019) was 16.7, rebounding post-pandemic with 13 cases in 2021, followed by 12 in 2022, 18 in 2023, and a peak of 21 cases in 2024. These fluctuations underscore how external factors such as public health policy and rink accessibility can influence injury rates-and by extension, the seasonal workload for orthopaedic and hand surgery teams.

Poisson regression (Table 2) revealed no significant year-on-year trend (IRR per year = 1.01, 95% CI 0.94-1.09, p = 0.73), but confirmed a statistically significant reduction in the 2020 season (IRR = 0.19, 95% CI 0.06-0.58, p = 0.004). Although observational, these findings support the notion that injury volumes from temporary ice rinks represent a predictable seasonal burden, one that trauma services can anticipate and plan for during peak months of operation.

**Table 2:** Poisson regression analysis of annual ice rink-related hand and wrist injury incidence (2017–2024) *COVID-19 season defined as the 2020 ice skating season (November 2020 to January 2021). Data are presented as incidence rate ratios (IRRs) with 95% confidence intervals. Statistical significance was defined as p < 0.05.

Predictor	IRR	95% CI	p-value
Year (continuous)	1.01	0.94 – 1.09	0.73
COVID-19 season*	0.19	0.06 – 0.58	0.004

## Discussion

The temporary ice rink near our major trauma centre operates for approximately 10 to 11 weeks each winter season, generally from early November until late January. During this period, the rink is reported to schedule around 6-8 public skating sessions per day, which amounts to approximately 42 to 56 sessions each week. Each of these sessions can accommodate roughly 150 to 200 skaters at a time, depending on ticket sales and operational capacity [12]. With a maximum capacity of 150-200 skaters per session, the total seasonal attendance at full capacity would be approximately 90,000-95,000 skater-visits. However, assuming a more conservative average occupancy of 75%, the estimated seasonal attendance would be closer to 67,500-71,250 skater-visits. Based on our data, which identified an average of 15 bony injuries per season, this equates to an incidence of approximately 2.1 injuries per 10,000 skater-visits at 75% occupancy - and even at full capacity, the incidence remains at around 1.7 per 10,000 skater-visits. Although this rate is relatively low when considered per visit, it represents a clinically meaningful burden for local trauma services, particularly given the potential severity and need for operative management in some cases.

This eight-year review highlights the burden of ice rink-related hand injuries presenting to a major trauma centre, with a clear predominance of distal radius fractures, which comprised 67.5% of bony injuries in our cohort, and a gradual but notable surge in injury numbers in the later years of the study period. Our findings are consistent with existing literature demonstrating the vulnerability of the upper limb - particularly the distal radius - during falls on ice rinks, a pattern often attributed to falls on outstretched hands and compounded by limited protective equipment use [3,6,7,9]. Observational work at other UK temporary rinks has similarly reported a high proportion of upper limb fractures, particularly involving the wrist, consistent with our predominance of distal radius injuries [13]. The high rate of operative fixation, particularly open reduction and internal fixation, underscores the potential severity of these injuries, and the demands placed on orthopaedic and hand surgery services during rink operation periods.

The gradual increase in injury numbers in the most recent years may reflect changing patterns of rink use, increased public participation following periods of pandemic restrictions, or local environmental factors. Similar seasonal and episodic surges have been documented in other settings, particularly when natural or temporary rinks attract large crowds without commensurate safety infrastructure [1,2,4].

Advanced imaging played an important role in diagnostic evaluation, with MRI and CT scans used to clarify occult fractures and articular involvement. This finding aligns with prior recommendations for careful diagnostic workup given the frequent under-detection of scaphoid or intra-articular injuries on plain radiographs [14].

Importantly, our data reflect injuries sustained at a single temporary rink in an urban UK setting. Previous work has emphasised the risks specific to temporary rinks, including poor surface maintenance, high user density, and limited use of protective barriers [1,2]. These environmental factors, combined with the demographic profile of skaters - often casual participants without formal training - may explain the predominance of high-energy falls onto outstretched hands.

Our findings also align with broader literature describing the impact of temporary ice rinks on local healthcare services. Multiple studies have documented increased attendances and surges in emergency presentations during rink operation periods, highlighting the seasonal strain on hospitals and orthopaedic teams [15,16]. Moreover, Schwarzkopf et al. have emphasised the hidden costs of such injuries, noting that even ‘free’ or low-cost public skating facilities can create a substantial downstream healthcare burden that underscores the importance of planning and prevention [17].

Despite calls for injury prevention strategies, including rink design improvements, blade guards, and public education [1,2,10], our study suggests that a significant hand injury burden persists. Given the substantial proportion of injuries requiring surgery and follow-up care, these data reinforce the need for preventive measures and targeted public health messaging.

Strengths and limitations

This study has both strengths and limitations that should be considered when interpreting the findings. Among its strengths, it represents one of the longest continuous reviews of ice rink-related hand and wrist injuries in the literature, spanning eight consecutive skating seasons. The use of a prospectively maintained VFC database ensured consistent documentation and reliable case capture. By focusing on a major trauma centre located adjacent to a large seasonal ice rink, the study provides an accurate reflection of the predictable burden such facilities place on local trauma and hand surgery services. The detailed analysis of injury patterns, imaging utilisation, and operative demand offers clinically relevant insights for service planning. Furthermore, the use of Poisson regression allowed a robust evaluation of temporal trends, including the effect of the COVID-19 pandemic, adding a novel longitudinal perspective that has been lacking in prior reports.

Nonetheless, several limitations should be acknowledged. First, only patients referred through the VFC pathway were included; individuals discharged directly from the emergency department without hand service involvement were not captured, which may have led to an underestimation of the true incidence of ice rink-related injuries. Second, the classification of cases as ice rink-related relied on the recorded patient history at presentation, which is susceptible to reporting or recording bias. Third, as a single-centre study, the findings may not be generalisable to other hospitals or regions with different rink conditions, safety measures, or referral practices. Fourth, although the dataset spanned eight years, the sample size was modest, limiting the ability to detect smaller trends. Finally, the absence of exposure data, such as individual skating experience, protective equipment use, or rink attendance, restricted the ability to calculate precise incidence rates per participant. Despite these limitations, the study provides valuable longitudinal evidence to inform both clinical service planning and injury prevention strategies.

Recommendation

Effective strategies to reduce ice rink-related hand injuries should begin with robust public health education that raises awareness of the specific risks associated with falls onto outstretched hands and promotes safer skating behaviours [1,2,10].

For instance, the use of protective equipment such as wrist guards and padded gloves should be actively encouraged, drawing on evidence from other skating sports where such measures have been shown to reduce the incidence of distal radius fractures [2,6,10]. Collaboration with local authorities and rink operators is essential to improve rink safety by ensuring consistent surface maintenance, managing crowd density, and considering design enhancements such as barriers and padding to minimise fall-related injuries [1,2]. Additionally, trauma and hand surgery services should anticipate and plan for seasonal increases in case volume during rink operation periods, with particular attention to imaging resources and surgical capacity. Finally, there is a clear need for future prospective, multicentre research to evaluate the effectiveness of preventive strategies and to better characterise injury epidemiology across diverse rink settings [1,4,10].

## Conclusions

Temporary ice rink-related hand and wrist injuries remain a predictable seasonal burden on trauma services, with distal radius fractures accounting for the majority of cases and contributing substantially to the surgical workload. These injuries frequently require advanced imaging and operative management, reflecting their clinical severity. The observed reduction during the COVID-19 pandemic and subsequent rebound highlight the influence of public participation and seasonal trends. Preventive strategies, including public education, promotion of protective equipment, and improved rink safety standards, are essential. Anticipatory service planning and further multicentre research are warranted to evaluate preventive measures and reduce the healthcare burden.
